# Combined phacoemulsification and angle filtering procedures versus phacoemulsification with clinical outcomes in primary glaucoma coexisting with cataracts: a meta-analysis of randomized controlled trials

**DOI:** 10.3389/fopht.2026.1787044

**Published:** 2026-06-08

**Authors:** Tangshou Xie, Wenrong Rao, Xi Liu, Yuanyi Li, Jie Zhu

**Affiliations:** 1 Nanping First Hospital Affiliated to Fujian Medical University, Nanping, Fujian, China; 2 Nanping Peoples’ Hospital, Nanping, Fujian, China

**Keywords:** cataract, glaucoma, meta-analysis, phacoemulsification, phacotrabeculectomy

## Abstract

**Background:**

To evaluate the clinical outcomes of combined phacoemulsification and angle filtering procedures versus phacoemulsification alone in patients with primary glaucoma coexisting with cataracts.

**Methods:**

Randomized controlled trials comparing combined phacoemulsification and angle filtering procedures with phacoemulsification published before September 24, 2025, were identified through PubMed/Medline, Embase, and Cochrane Library. Intraocular pressure (IOP), anti-glaucoma medications (AGMs), best-corrected visual acuity (BCVA), perimetry, and complications were analyzed in this meta-analysis. Subgroup analyses were conducted based on follow-up duration, angle status, use of mitomycin C (MMC), and surgical approach.

**Results:**

Fourteen randomized controlled trials were pooled for analysis. Combined phacoemulsification and angle filtering procedures had a smaller effect on BCVA than phacoemulsification alone without MMC [mean difference (MD) = 0.07, confidence interval (CI): 0.01 to 0.13, I^2^ = 49%, p = 0.03]. Combined phacoemulsification and angle filtering procedures were significantly more effective in reducing IOP (MD = −1.98, CI: −2.71 to −1.25, I^2^ = 90%, p < 0.0001) and need of AGMs (MD = −0.69, CI: −0.87 to −0.52, I^2^ = 73%, p < 0.0001) but associated with a significantly higher rate of complications [risk ratio (RR) = 2.66, CI: 1.82 to 3.89, I^2^ = 13%, p < 0.00001].

**Conclusions:**

Combined phacoemulsification and angle filtering procedures are superior in reducing IOP and need for AGMs. In the subgroup without MMC use, combined angle filtering procedures were associated with a statistically significant difference in BCVA compared with phacoemulsification alone.

**Systematic review registration:**

https://inplasy.com/inplasy-2026-5-0081/, identifier INPLASY202650081.

## Introduction

In recent decades, combined phacoemulsification and angle filtering procedures have been widely performed in glaucoma surgery, which consists of extirpation of the lens, intraocular lens implantation, and trabeculectomy in one procedure, aimed at removing lens obstacles, improving visual acuity, and decreasing intraocular pressure (IOP). In contrast, phacoemulsification involves only the extirpation of the lens and intraocular lens implantation. Cataract extraction itself is known to be a safe and effective surgery that can lower IOP, although the specific mechanisms for this effect are still unclear (1). Compared with derange implants, simple filter procedures are easier to implement. We have a growing interest in the combined phacoemulsification and angle filtering procedure because one more step may not only have a better clinical outcome but also cause severe complications.

This expansion in surgical options highlights the need for clear evidence of their efficacy to guide surgeons in selecting the most appropriate technique. To our knowledge, no meta-analysis has analyzed the clinical outcomes to compare combined phacoemulsification and angle filtering procedures versus phacoemulsification alone. Therefore, this meta-analysis was conducted to compare the clinical outcomes between combined phacoemulsification and angle filtering procedures and phacoemulsification in primary glaucoma coexisting with cataracts.

## Materials and methods

### Research strategy

“Phacotrabeculectomy” was defined as including all phacoemulsification and angle filtering procedures. The deadline for screening published articles was September 24, 2025. MeSH terms included “glaucoma” and “cataract”. Published articles reporting “phacoemulsification” were screened. Similarly, “trabectome”, “ab interno”, “ab externo”, “triple procedure”, and “phacotrabeculectomy” were incorporated into the search strategy. Relevant studies were identified through a literature search in the following databases: PubMed/Medline, Embase, and Cochrane Library. The complete PubMed search strategy is provided in the **Supplementary Material**. Reference lists of included studies were also manually reviewed to identify additional eligible articles. Two reviewers (X. Liu and Y. Li) independently screened studies, with a third reviewer (T. Xie) adjudicating discrepancies.

### Selection criteria

Inclusion criteria were as follows: 1) patients diagnosed with primary open-angle glaucoma, angle-closure glaucoma, or primary angle-closure; 2) studies comparing surgical techniques, including combined phacoemulsification and angle filtering procedures as the experimental group and phacoemulsification as the control group; 3) clinical outcomes including IOP, anti-glaucoma medications (AGMs), complications, best-corrected visual acuity (BCVA), and perimetry; and 4) randomized controlled trials only.

Exclusion criteria were as follows: 1) articles that did not involve trabeculectomy and phacoemulsification; 2) articles with data merged from a published search by the same research team; 3) studies including other types of glaucoma, such as secondary glaucoma, traumatic glaucoma, or congenital glaucoma; and 4) patients with a history of prior ocular surgery (including laser peripheral iridotomy, selective laser trabeculoplasty, or other ocular surgical procedures).

### Outcome statistical analysis

IOP, AGMs, BCVA, and perimetry outcomes were presented as mean difference (MD). Complications were presented as risk ratios (RRs). A random-effects model was used in this analysis. Heterogeneity between studies was assessed using statistics, and subgroup analysis was used to minimize heterogeneity. The confidence interval (CI) was 95%. Publication bias was evaluated using RevMan funnel plots. A p-value of <0.05 was considered statistically significant. All data analyses were conducted using RevMan 5.0. Publication bias was assessed using Egger’s test in STATA.

## Results

**Figure 1:**
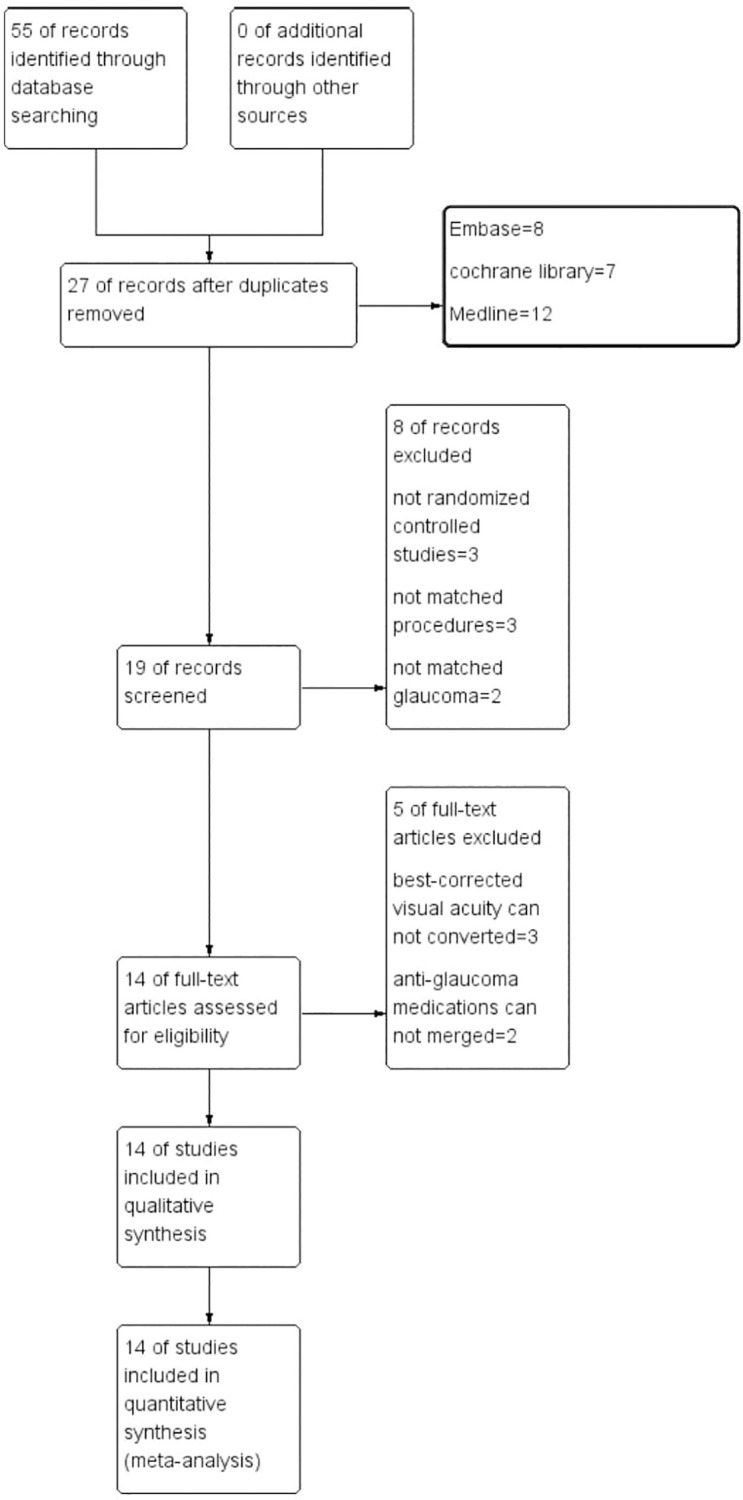
Preferred Reporting Items for Systematic reviews and Meta-Analyses (PRISMA) flow diagram using RevMan 5.0.

**Figure 2:**
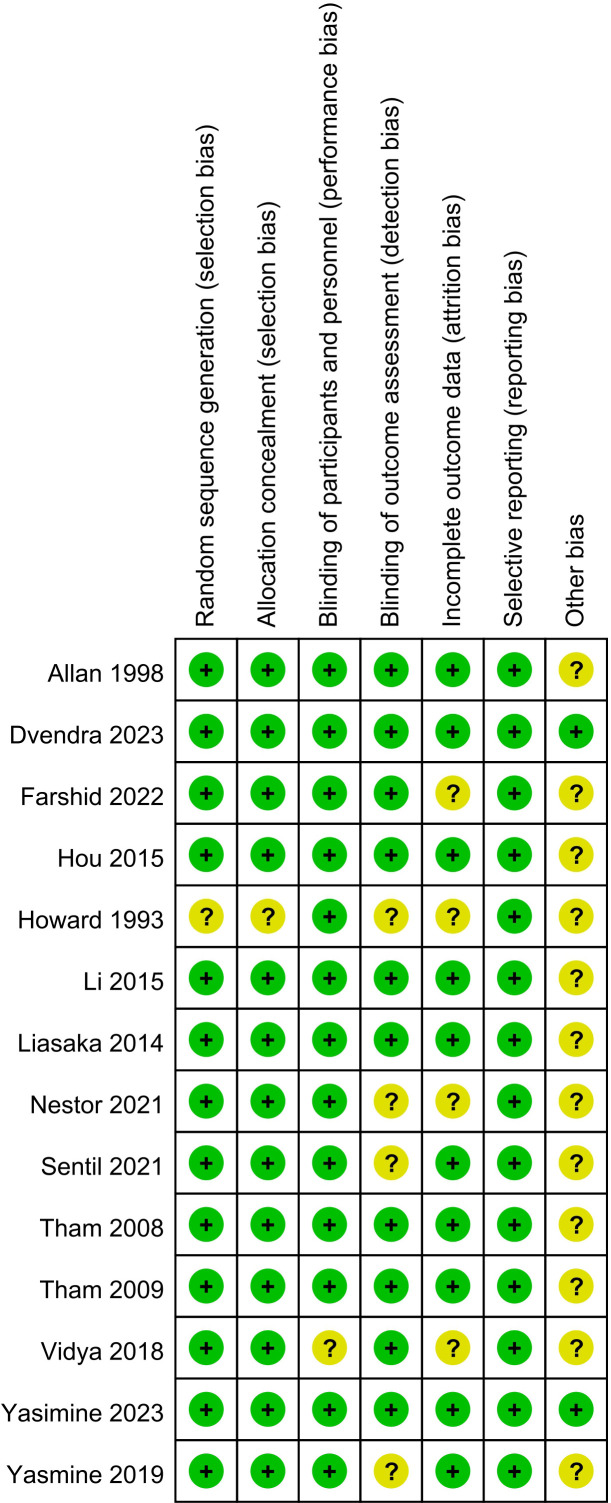
Risk of bias summary of included studies.

A total of 55 studies were identified through database searching. This study applied no language restrictions. Duplicate studies and articles involving glaucoma drainage implants were excluded. The flow diagram is shown in **Figure 1**. Finally, 14 studies were pooled for the final meta-analysis (2–15). All 14 randomized controlled trials diagnosed angle open or not by gonioscopy. The characteristics of included studies are shown in **Table 1**, and the quality assessment of included studies is shown in **Figure 2**. Due to insufficient data, perimetry analysis was not performed. Only one study was conducted in uncontrolled primary angle-closure glaucoma (PACG) (5). Only one study was conducted in advanced primary open-angle glaucoma (POAG) (7). Subgroup analysis based on disease stage and control status was also not performed. For each outcome, subgroup analysis was used to minimize heterogeneity.

**Table 1:** Characteristics of included studies.

Author	Region	Year	Age		Cases		Glaucoma	Procedure		Finish follow-up	
			Phacotrab^i^	Phaco^ii^	Phacotrab	Phaco		Phacotrab	Phaco	Phacotrab	Phaco
**Yasmine** (2)	Eygpt	2019	57.3±8.3	58.8±8.4	31	32	PACG^iii^	phacotrab+MMC	Phaco	16.8±7.5(m^iv^)	20.4±6.5(m)
**Hou** (3)	China	2015	62.38±9.45	62.32±8.48	24	25	PACG	phacotrab+MMC	Phaco	12(m)	12(m)
**Allan** (4)	Denmark	1998	57-83	77-88	10	10	POAG	Phacotrab	Phaco	12(m)	12(m)
**Tham** (5)	China	2008	71.4±6.6	71.9±6.7	37	35	PACG	phacotrab+MMC^v^	Phaco	30.6±5.9(m)	30.9±8.4(m)
**Tham** (6)	China	2009	70.4±9.4	70.3±7.4	24	27	PACG	phacotrab+MMC	Phaco	37.6±10.3(m)	33.4±10.5(m)
**Liaska** (7)	Athens	2014	77.0±6.7	78.1±7.26	29	31	POAG	phacotrab+MMC	Phaco	24(m)	24(m)
**Vidya** (8)	Saudi Arabia	2018	61.9±5.7	63.7±5.9	45	46	PACG	Phacotrab	Phaco	12(m)	12(m)
**Howard** (9)	Canada	1993	75.5	77.5	51	51	POAG^vi^	Phacotrab	Phaco	24(m)	24(m)
**Senthil** (10)	India	2021	58.45±9.8	61.6±8.9	37	33	PACG	Phacotrab	Phaco	2.5±1.8(y^vii^)	2.8±2.0(y)
**Nestor** (11)	Spain	2021	79.4±6.8	78.5±6.1	21	21	POAG	Phacotrab	Phaco	12(m)	12(m)
**Farshid** (13)	Iran	2022	59.1±6.1	59.4±5.7	27	25	PACG	Phacotrab	Phaco	6(m)	6(m)
**Yasmine** (14)	Eygpt	2023	60.7±9.58	62.6±10.45	36	38	PACG	Phacotrab	Phaco	12(m)	12(m)
**Dvendra** (15)	India	2023	62.2±8.4	63.1±8.2	57	57	POAG	Phacotrab	Phaco	12(m)	12(m)
**Li** (12)	China	2015	69.5±9.12	69.5±9.12	31	31	PACG	Phacotrab	Phaco	6(m)	6(m)

i Phacotrab: Phacotrabeculectomy group;

ii Phaco: Phacoemulsification group

iii PACG: Primary angle-closure glaucoma

iv m: month

v Phacotrab+MMC: Phacotrabeculectomy+mitomy C

vi POAG: Primary open angle glaucoma

vii y: year

### BCVA

A total of nine studies were pooled in this analysis (2, 3, 5–7, 10, 12–14). A random-effects model was used. Phacoemulsification showed improved outcome on BCVA compared with combined phacoemulsification and angle filtering procedures (MD=0.05, CI: 0.01 to 0.08, I^2^ = 0.0%, p = 0.008).

**Figure 3:**
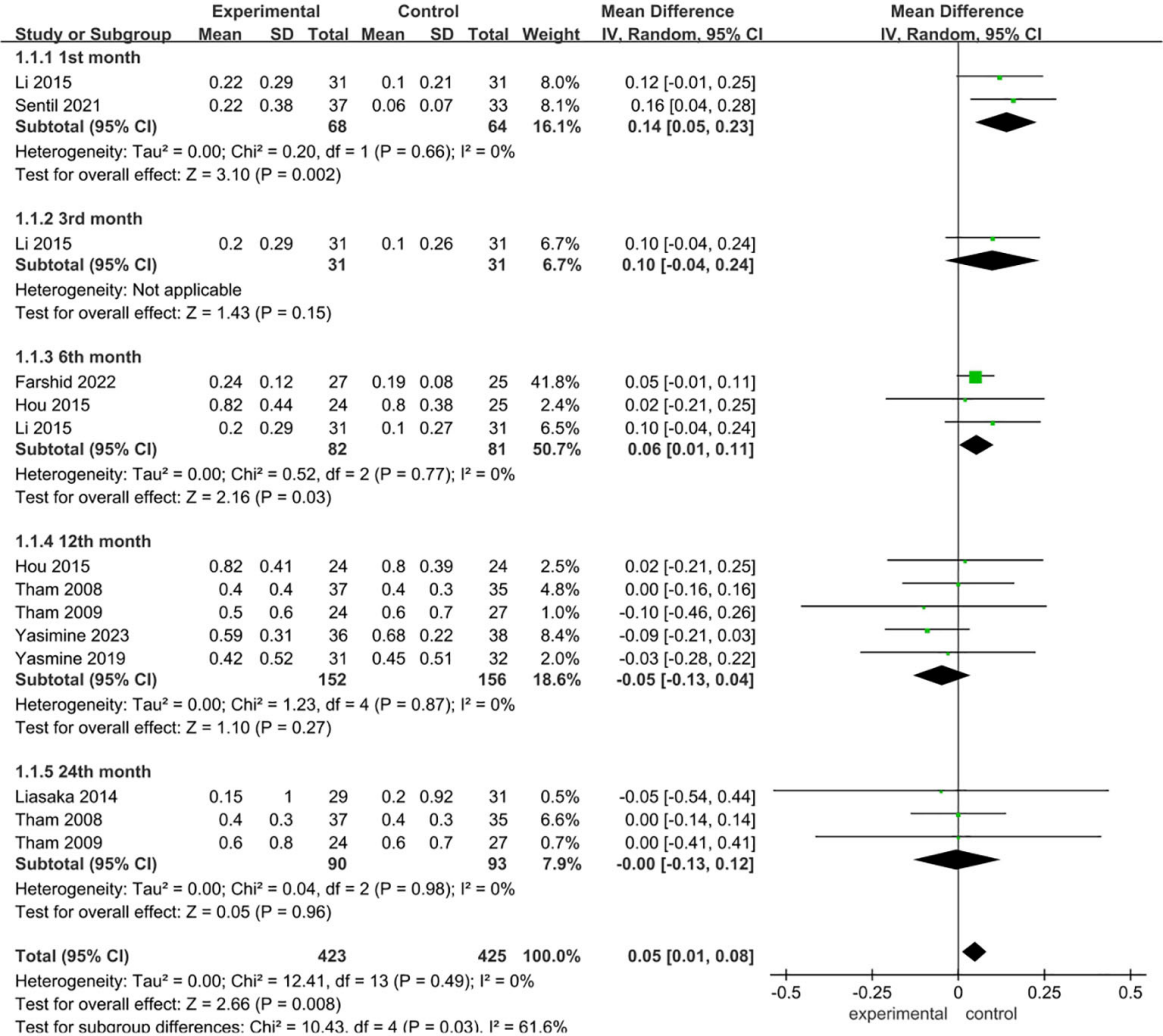
Forest plot of best-corrected visual acuity (logMAR) by follow-up duration.

Subgroup analysis was conducted based on follow-up time. In the first month (MD=0.14, CI: 0.05 to 0.23, I^2^ = 0.0%, p = 0.002) and sixth month (MD=0.06, CI: 0.01 to 0.11, I^2^ = 0.0%, p = 0.03), combined phacoemulsification and angle filtering procedures were inferior to phacoemulsification. However, no difference was found between the two groups at the 12th month (MD = −0.05, CI: −0.13 to 0.04, I^2^ = 0.0%, p = 0.27) and 24th month (MD=0.00, CI: −0.13 to 0.12, I^2^ = 0.0%, p = 0.96). The forest plot of BCVA by follow-up is shown in **Figure 3**.

Second, subgroup analysis was conducted comparing PACG and POAG. Combined phacoemulsification and angle filtering procedures showed no significant difference in BCVA compared with phacoemulsification (MD=0.00, CI: −0.13 to 0.13, I^2^ = 0.0%, p = 1.00) in PACG. Subgroup analysis of POAG was not possible, as only one study reported relevant data.

Third, subgroup analysis of BCVA was also performed based on whether mitomycin C (MMC) was used. In the subgroup without MMC, combined phacoemulsification and angle filtering procedures had an inferior effect on BCVA compared to phacoemulsification (MD=0.07, CI: 0.01 to 0.13, I^2^ = 49%, p = 0.03). However, the two procedures had the same effect in the subgroup with MMC (MD=0.00, CI: −0.08 to 0.08, I^2^ = 0.0%, p = 0.97).

In the subgroup analysis stratified by MMC use, among patients who did not use MMC, the combined phacoemulsification and angle filtering group showed a statistically significant but small difference in BCVA compared with the phacoemulsification alone group (MD=0.07, 95% CI: 0.01 to 0.13, Z=2.12, p = 0.03). Heterogeneity was moderate and not statistically significant (I^2^ = 49%, Tau^2^ = 0.00, Chi^2^ = 9.81, df = 5, p = 0.08).

Fourth, subgroup analysis of BCVA was performed based on surgical approach (ab interno or ab externo). Because the subgroup ab interno and subgroup ab externo had data for analysis just at the 12th-month follow-up point, subgroup analysis was conducted at this time point. Two procedures had the same effect in the subgroup ab externo (MD = −0.01, CI: −0.12 to 0.10, I^2^ = 0.0%, p = 0.95). Subgroup analysis of ab interno was not possible because only one study reported relevant data.

### IOP

A total of 12 studies were included in the analysis (2–7, 9, 11–15). A random-effects model was used. Combined phacoemulsification and angle filtering procedures presented superior results on lowering IOP compared with phacoemulsification (MD = −1.98, CI: −2.71 to −1.25, I^2^ = 90%, p < 0.0001).

**Figure 4:**
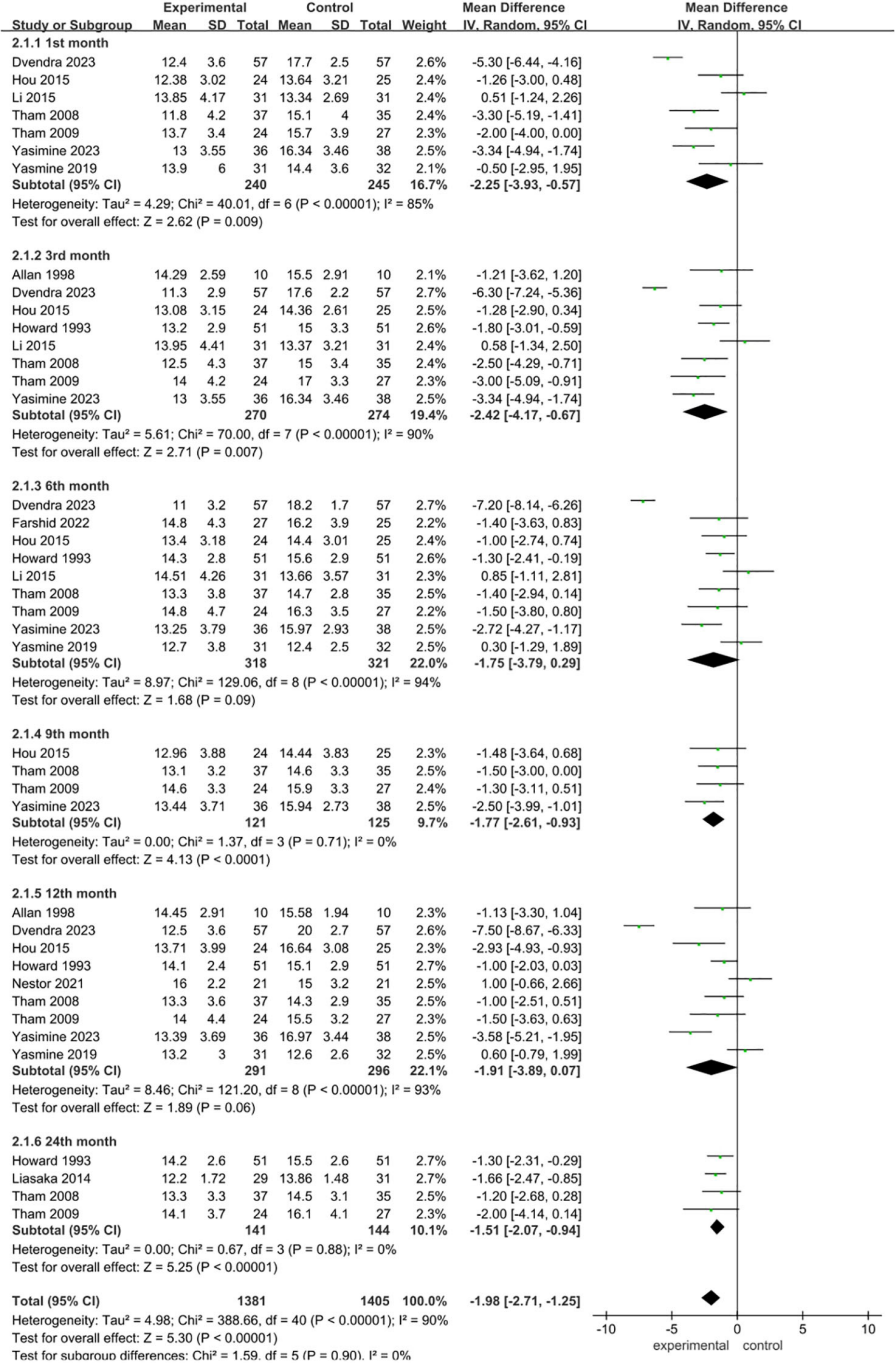
Forest plot of intraocular pressure (mmHg) by follow-up duration.

First, subgroup analysis was conducted based on follow-up time. The combined phacoemulsification and angle filtering group exhibited significantly lower IOP at first-month, third-month, ninth-month, and 24th-month follow-up time points, and the results were (MD = −2.25, CI: −3.93 to −0.57, I^2^ = 85%, p = 0.009), (MD = −2.42, CI: −4.17 to −0.67, I^2^ = 90%, p = 0.007), (MD = −1.77, CI: −2.61 to −0.93, I^2^ = 0.0%, p < 0.0001), and (MD = −1.51, CI: −2.07 to −0.94, I^2^ = 0%, p < 0.0001), respectively. IOP in the sixth month (MD = −1.75, CI: −3.79 to 0.29, I^2^ = 94%, p = 0.09) and 12th month (MD = −1.91, CI: −3.89 to 0.07, I^2^ = 93%, p = 0.06) showed no difference. The forest plot of IOP by follow-up is shown in **Figure 4**.

Second, subgroup analysis comparing PACG versus POAG was also conducted, indicating that the combined phacoemulsification and angle filtering procedures significantly lowered IOP in both PACG (MD = −1.78, CI: −2.26 to −1.31, I^2^ = 41%, p < 0.0001) and POAG (MD = −2.97, CI: −4.69 to −1.25, I^2^ = 95%, p = 0.0007).

Third, IOP was also analyzed according to the use of MMC. Combined phacoemulsification and angle filtering procedures significantly lowered IOP in both the subgroup without MMC (MD = −2.44, CI: −3.69 to −1.20, I^2^ = 94%, p = 0.0001) and the subgroup with MMC (MD = −1.39, CI: −1.80 to −0.99, I^2^ = 16%, p < 0.0001). The pooled IOP analysis showed substantial heterogeneity (I^2^ = 90%, p < 0.001). Subgroup analysis stratified by MMC use revealed a marked reduction in heterogeneity with the MMC subgroup (I^2^ = 16%, p = 0.25), whereas heterogeneity remained high in the subgroup without MMC (I^2^ = 94%, p < 0.001).

Fourth, subgroup analysis of IOP was performed based on surgical approach (ab interno or ab externo). Phacoemulsification and angle filtering procedures significantly lowered IOP in both the subgroup ab interno (MD = −4.14, CI: −5.62 to −2.60, I^2^ = 93%, p < 0.001) and subgroup ab externo (MD = −1.16, CI: −1.55 to −0.77, I^2^ = 28%, p = 0.03).

### AGMs

A total of seven studies were included in this analysis (2, 4–7, 13, 15). A random-effects model was applied. Combined phacoemulsification and angle filtering procedures showed better outcomes by decreasing IOP compared with phacoemulsification (MD = −0.69, CI: −0.87 to −0.52, I^2^ = 73%, p < 0.0001).

**Figure 5:**
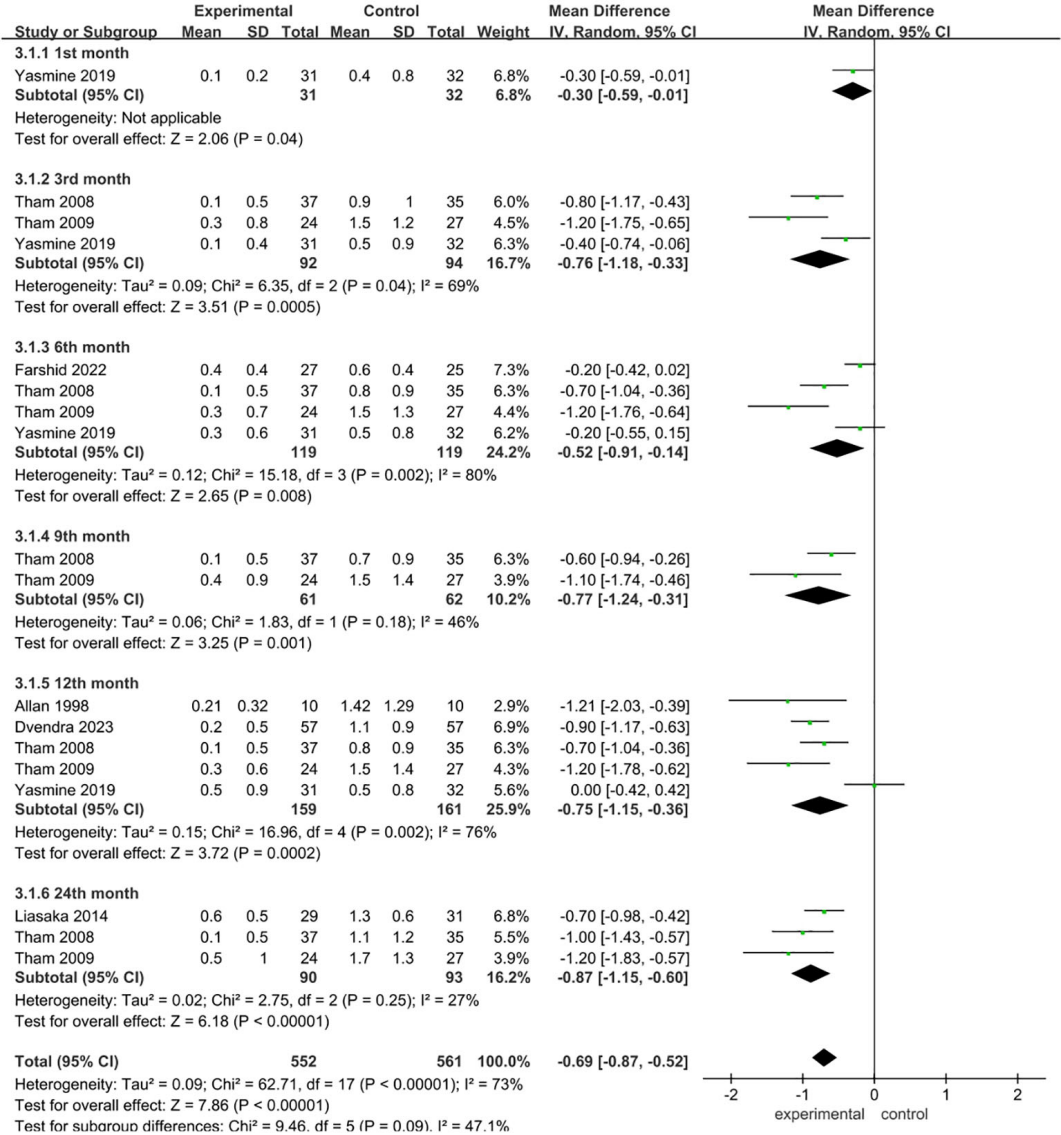
Forest plot of anti-glaucoma medications by follow-up duration.

First, subgroup analysis was conducted based on follow-up time. The combined surgery group exhibited significantly fewer AGMs at all follow-up time points. The results were (MD = −0.3, CI: −0.59 to −0.01, p = 0.04), (MD = −0.76, CI: −1.18 to −0.33, I^2^ = 69%, p = 0.0005), (MD = −0.52, CI: −0.91 to −0.14, I^2^ = 80%, p = 0.008), (MD = −0.77, CI: −1.24 to −0.31, I^2^ = 46%, p = 0.001), (MD = −0.75, CI: −1.15 to −0.36, I^2^ = 76%, p = 0.0002), and (MD = −0.87, CI: −1.15 to −0.60, I^2^ = 27%, p < 0.00001). The forest plot of AGMs by follow-up is shown in **Figure 5**.

Second, subgroup analysis comparing PACG to POAG was also conducted. AGMs were significantly fewer in the combined phacoemulsification and angle filtering procedures group in both the PACG group (MD = −0.79, CI: −1.22 to −0.35, I^2^ = 77%, p = 0.0004) and the POAG group (MD = −0.83, CI: −1.01 to −0.64, I^2^ = 0.0%, p < 0.00001).

Third, AGMs were also analyzed according to MMC use. Both procedures resulted in fewer AGMs, either without MMC (MD = −0.70, CI: −1.30 to −0.10, I^2^ = 89%, p = 0.02) or with MMC (MD = −0.63, CI: −0.98 to −0.28, I^2^ = 77%, p = 0.0004).

Fourth, subgroup analysis of AGMs was also performed based on surgical approach (ab interno or ab externo). AGMs were significantly fewer in the combined phacoemulsification and angle filtering procedures group in the subgroup ab externo (MD = −0.72, CI: −1.27 to −0.18, I^2^ = 77%, p = 0.0004). Subgroup analysis of ab interno was not possible, as only one study reported relevant data.

### Perimetry

No statistics from the included studies could be pooled for perimetry analysis. Four studies recorded perimetry in their results. One study demonstrated enhanced perimetry mean deviation in the combined phacoemulsification and angle filtering procedures group compared to the phacoemulsification group at 24 months of follow-up (7). The remaining three studies (5, 6, 10) recorded no difference between the two groups preoperatively and postoperatively.

### Vertical cup disc ratio

Only two articles recorded the vertical cup-to-disc ratio, both using the same dataset from the same corresponding author (5, 6). The vertical cup-to-disc ratio was similar between the two groups from preoperatively to the 12th-month and 24th-month follow-up points.

### Complications

**Figure 6:**
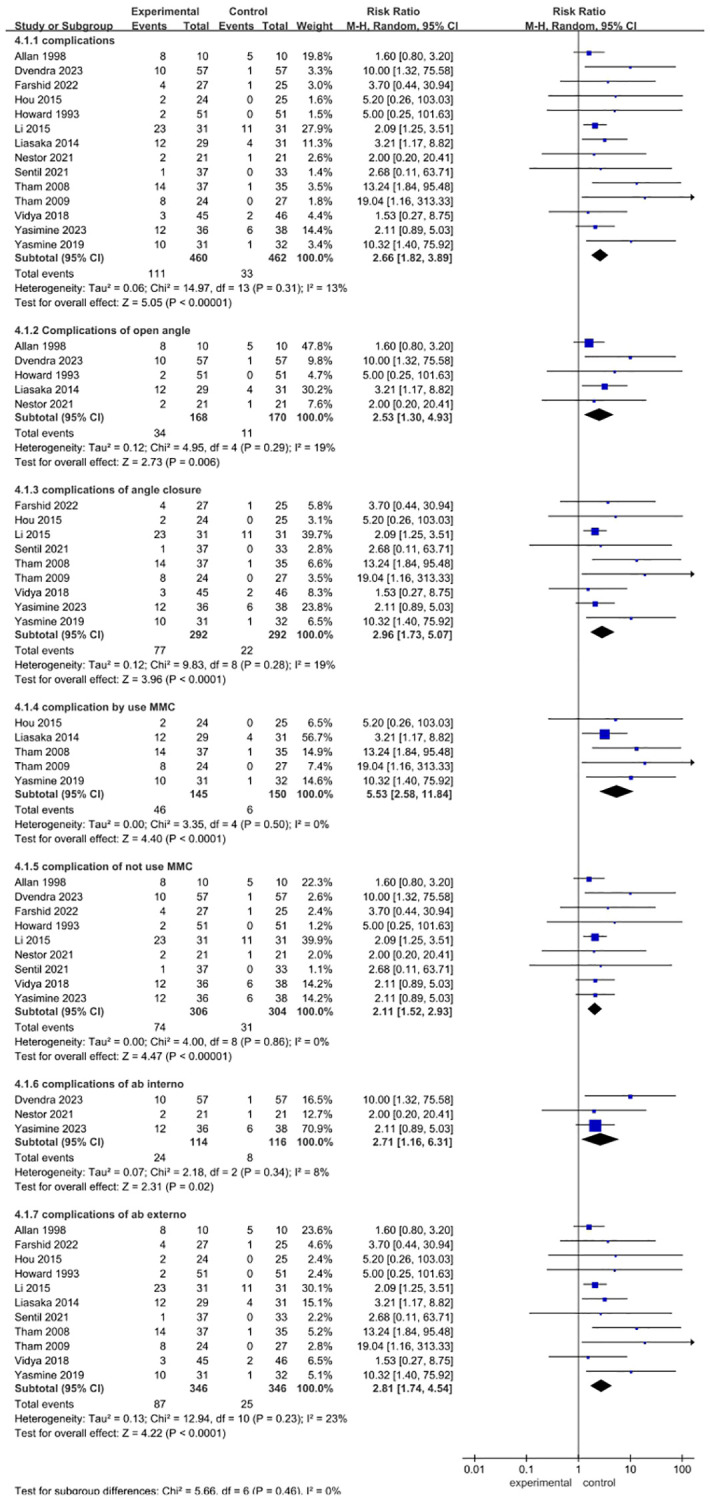
Forest plot of incidence of complications.

A total of 14 studies were included in this analysis (2–15). Risk ratio was used, and a random-effects model was applied. The forest plot of complications is shown in **Figure 6**. Combined phacoemulsification and angle filtering procedures showed a higher rate of complications compared with phacoemulsification (RR=2.66, CI: 1.82 to 3.89, I^2^ = 13%, p < 0.00001).

Subgroup analyses revealed that the combined phacoemulsification and angle filtering procedures group had a higher rate of complications in both the PACG group (RR=2.96, CI: 1.73 to 5.07, I^2^ = 19%, p < 0.0001) and the POAG group (RR=2.53, CI: 1.30 to 4.93, I^2^ = 19%, p = 0.006).

Third, complications were also analyzed based on the use of MMC. The combined phacoemulsification and angle filtering procedures group had a higher rate of complications in both the subgroup without MMC (RR=2.11, CI: 1.52 to 2.93, I^2^ = 0.0%, p < 0.00001) and the subgroup with MMC (RR=5.53, CI: 2.58 to 11.84, I^2^ = 0.0%, p < 0.0001).

Fourth, complications were also analyzed according to surgical approach (ab interno or ab externo). The combined phacoemulsification and angle filtering procedures group had a higher rate of complications in both the subgroup ab interno (RR=2.71, CI: 1.16 to 6.31, I^2^ = 8%, p = 0.02) and the subgroup ab externo (RR=2.81, CI: 1.74 to 4.54, I^2^ = 23%, p < 0.0001).

In the combined procedures group, postoperative IOP spikes were the most frequent complication, followed by microcystic changes. Hypotony and spontaneous choroidal detachment were less common, while malignant glaucoma and macular edema were rare. In the phacoemulsification group, an IOP ≥ 21 mmHg was the most frequently observed complication.

### Publication bias

All Egger’s tests yielded p > 0.05. No statistical evidence of publication bias was detected across any outcome.

The intercept of BCVA was non-significant (p = 0.262). However, the test was statistically underpowered due to the small number of included studies. With five studies, Egger’s test had unacceptably low statistical power to detect genuine publication bias. These results should be interpreted with extreme caution and considered exploratory only.

For both time points, Egger’s regression tests of IOP yield non-significant intercepts (p = 0.291 and p = 0.511). The null hypothesis of no funnel plot asymmetry was retained.

Although studies fell below the conventional threshold for optimal test power, the consistent non-significant results across both time points supported the absence of small-study effects.

The intercept of AGMs was non-significant (p = 0.221). As with BCVA, the limited number of studies severely restricted the validity of this test. This analysis was exploratory only and cannot provide reliable evidence regarding the presence or absence of publication bias.

The null hypothesis of no small-study effects (intercept = 0) was not rejected at the α = 0.05 significance level. There was insufficient statistical evidence to suggest publication bias or funnel plot asymmetry for the complications outcome. With 14 studies, the test had adequate statistical power. The positive intercept (1.3827) suggested a non-significant trend toward asymmetry, although not reaching conventional statistical significance.

Results for outcomes with the number of studies fewer than 10 (BCVA and AGMs) should be interpreted with caution due to insufficient statistical power. Publication bias of BCVA, IOP, AGMs, and complications was also assessed using funnel plots, which are presented in the **Supplementary Material**.

## Discussion

This is the first meta-analysis comparing the clinical outcomes between combined phacoemulsification and angle filtering procedures and phacoemulsification in primary glaucoma.

This meta-analysis demonstrated that combined phacoemulsification and angle filtering procedures are superior to phacoemulsification in reducing IOP and the need for anti-glaucoma medications. Seven studies also support the superiority of combined phacoemulsification and angle filtering procedures in decreasing IOP and using fewer AGMs (2, 7, 9, 11–14). Subgroup analysis suggested that MMC use was a major contributor to heterogeneity in IOP outcomes, as evidenced by the substantial reduction in I^2^ from 90% to 16% after stratification. However, the persistent high heterogeneity in the subgroup without MMC (I^2^ = 94%) indicates the presence of additional unidentified sources of heterogeneity.

BCVA is one of the major reasons for performing combined phacoemulsification and angle filtering procedures as a combined procedure (16). A randomized controlled trial conducted in advanced POAG showed that combined phacoemulsification and angle filtering procedures had the same visual outcome as phacoemulsification (7). In contrast, a randomized controlled trial conducted in PACG showed better visual outcomes in the phacoemulsification group (12). These findings suggest that visual acuity may not be an appropriate primary index for managing glaucoma progression. Similarly, our study indicated that combined phacoemulsification and angle filtering procedures resulted in better BCVA.

Our study showed that, in the subgroup without MMC, combined phacoemulsification and angle filtering procedures had an inferior effect on BCVA.

Although the subgroup analysis without MMC use revealed a statistically significant difference in BCVA favoring phacoemulsification alone (MD=0.07, p = 0.03), the effect size corresponded to approximately 3–4 Early Treatment Diabetic Retinopathy Study (ETDRS) letters, suggesting that this finding is unlikely to be of clinical relevance.

However, no difference in BCVA was observed when MMC was used. Considering the role of MMC in glaucoma filtering surgery, maybe early postoperative BCVA is affected by transient inflammation or bleb morphology. Optical coherence tomography (OCT) is a repeatable and useful imaging technique for evaluating and managing glaucoma (17), as it assesses the optic nerve and retinal nerve fiber layer thickness. Functional visual acuity has also been introduced to evaluate glaucoma patients. The concept of functional visual acuity has been considered applicable to detecting masked impairment of visual function in patients with dry eyes who complain of decreased visual acuity despite normal conventional visual acuity (18). Perhaps these methods can provide a more comprehensive analysis of abnormalities in visual outcomes from other perspectives.

The major complications observed in the two groups are summarized. In the combined phacoemulsification and angle filtering procedures group, postoperative IOP spikes were the most frequent complication, followed by microcystic changes. Hypotony and spontaneous choroidal detachment were the next most common, while malignant glaucoma and macular edema were rare. In the phacoemulsification group, IOP ≥ 21 mmHg occurred most frequently. Anti-metabolic chemicals such as MMC or 5-fluorouracil can reduce fibroblast collagen synthesis and inhibit cell migration and extracellular matrix production to control IOP (19). A higher rate of complications with the use of MMC occurred, which may be related to the timing and concentration of MMC. However, a review article highlighted the lack of uniform criteria for glaucoma procedures (20). To minimize complications caused by anti-metabolic chemicals, apart from drug concentration, IOP should be lowered preoperatively, and all tissues should be handled gently during surgery, such as using non-tooth, non-serrated forceps to minimize tissue trauma and bleeding (21).

Except in cases of glaucoma coexisting with diabetes, initial surgery is beneficial for glaucoma patients in perimetry monitoring (22). Both combined phacoemulsification and angle filtering procedures and phacoemulsification have favorable outcomes in postoperative perimetry (23). However, three included studies (5–7) recorded no significant difference between the two groups preoperatively and postoperatively. Two of these studies involved patients with advanced glaucoma to monitor perimetry, which may cause differences.

## Limitations

The findings of this study should be interpreted in light of several limitations. Although we analyzed POAG and PACG, other categories, such as secondary glaucoma and pseudoexfoliation glaucoma, still need to be evaluated. We did not analyze the data of aqueous humor drainage implants combined with phacoemulsification, although we conducted a subgroup analysis to minimize the heterogeneity. Heterogeneity can also be caused by other factors, such as differences in preoperative baseline data and surgical techniques. Additionally, this meta-analysis could not evaluate perimetry and retinal nerve fiber layer thickness due to insufficient data. Therefore, that means that our results and conclusions are insufficient.

## Conclusion

Combined phacoemulsification and angle filtering procedures are superior to phacoemulsification in reducing IOP and the need for AGMs but have a higher rate of complications. In the subgroup without MMC use, combined angle filtering procedures were associated with a statistically significant difference in BCVA compared with phacoemulsification alone.

## Data Availability

The original contributions presented in the study are included in the article/**Supplementary Material**. Further inquiries can be directed to the corresponding author.
