# Supplementary material for: Combined phacoemulsification and angle filtering procedures versus phacoemulsification with clinical outcomes in primary glaucoma coexisting with cataracts: a meta-analysis of randomized controlled trials
Source: Front Ophthalmol (Lausanne). 2026 Jun 8;6:1787044. doi: 10.3389/fopht.2026.1787044 (PMC13283822; doi:10.3389/fopht.2026.1787044)
Supplement: Supplementary file 1 [file SupplementaryFile1.docx]

| Query | Sort By | Filters | Search Details | Results | Time | Date |
| --- | --- | --- | --- | --- | --- | --- |
| (((Glaucoma[MeSH Terms]) OR (Glaucomas)) AND ((((((((((((Cataract[MeSH Terms]) OR (Cataracts)) OR (Lens Opacities)) OR (Lens Opacity)) OR (Opacities, Lens)) OR (Opacity, Lens)) OR (Cataract, Membranous)) OR (Cataracts, Membranous)) OR (Membranous Cataract)) OR (Membranous Cataracts)) OR (Pseudoaphakia)) OR (Pseudoaphakias))) AND ((Phacoemulsification) AND (((((Trabectome) OR (ab interno)) OR (ab externo)) OR (triple procedure)) OR (Phacotrabeculectomy))) | | | ("glaucoma"[MeSH Terms] OR ("glaucoma"[MeSH Terms] OR "glaucoma"[All Fields] OR "glaucomas"[All Fields] OR "glaucoma s"[All Fields])) AND ("cataract"[MeSH Terms] OR ("cataract"[MeSH Terms] OR "cataract"[All Fields] OR "cataracts"[All Fields] OR "cataractic"[All Fields] OR "cataractous"[All Fields]) OR ("cataract"[MeSH Terms] OR "cataract"[All Fields] OR ("lens"[All Fields] AND "opacities"[All Fields]) OR "lens opacities"[All Fields]) OR ("cataract"[MeSH Terms] OR "cataract"[All Fields] OR ("lens"[All Fields] AND "opacity"[All Fields]) OR "lens opacity"[All Fields]) OR ("cataract"[MeSH Terms] OR "cataract"[All Fields] OR ("opacities"[All Fields] AND "lens"[All Fields]) OR "opacities lens"[All Fields]) OR ("cataract"[MeSH Terms] OR "cataract"[All Fields] OR ("opacity"[All Fields] AND "lens"[All Fields]) OR "opacity lens"[All Fields]) OR ("cataract"[MeSH Terms] OR "cataract"[All Fields] OR ("cataract"[All Fields] AND "membranous"[All Fields]) OR "cataract membranous"[All Fields]) OR ("cataract"[MeSH Terms] OR "cataract"[All Fields] OR ("cataracts"[All Fields] AND "membranous"[All Fields])) OR ("cataract"[MeSH Terms] OR "cataract"[All Fields] OR ("membranous"[All Fields] AND "cataract"[All Fields]) OR "membranous cataract"[All Fields]) OR ("cataract"[MeSH Terms] OR "cataract"[All Fields] OR ("membranous"[All Fields] AND "cataracts"[All Fields]) OR "membranous cataracts"[All Fields]) OR ("cataract"[MeSH Terms] OR "cataract"[All Fields] OR "pseudoaphakia"[All Fields]) OR ("cataract"[MeSH Terms] OR "cataract"[All Fields])) AND (("phacoemulsification"[MeSH Terms] OR "phacoemulsification"[All Fields] OR "phacoemulsifications"[All Fields] OR "phakoemulsification"[All Fields]) AND ("Trabectome"[All Fields] OR (("abnormalities"[MeSH Subheading] OR "abnormalities"[All Fields] OR "ab"[All Fields]) AND "interno"[All Fields]) OR (("abnormalities"[MeSH Subheading] OR "abnormalities"[All Fields] OR "ab"[All Fields]) AND "externo"[All Fields]) OR (("triple"[All Fields] OR "triples"[All Fields]) AND ("methods"[MeSH Terms] OR "methods"[All Fields] OR "procedure"[All Fields] OR "methods"[MeSH Subheading] OR "procedures"[All Fields] OR "procedural"[All Fields] OR "procedurally"[All Fields] OR "procedure s"[All Fields])) OR ("phacotrabeculectomies"[All Fields] OR "phacotrabeculectomy"[All Fields]))) | 340 | 3:31:13 | 2025/10/19 |
| (Phacoemulsification) AND (((((Trabectome) OR (ab interno)) OR (ab externo)) OR (triple procedure)) OR (Phacotrabeculectomy)) | | | ("phacoemulsification"[MeSH Terms] OR "phacoemulsification"[All Fields] OR "phacoemulsifications"[All Fields] OR "phakoemulsification"[All Fields]) AND ("Trabectome"[All Fields] OR (("abnormalities"[MeSH Subheading] OR "abnormalities"[All Fields] OR "ab"[All Fields]) AND "interno"[All Fields]) OR (("abnormalities"[MeSH Subheading] OR "abnormalities"[All Fields] OR "ab"[All Fields]) AND "externo"[All Fields]) OR (("triple"[All Fields] OR "triples"[All Fields]) AND ("methods"[MeSH Terms] OR "methods"[All Fields] OR "procedure"[All Fields] OR "methods"[MeSH Subheading] OR "procedures"[All Fields] OR "procedural"[All Fields] OR "procedurally"[All Fields] OR "procedure s"[All Fields])) OR ("phacotrabeculectomies"[All Fields] OR "phacotrabeculectomy"[All Fields])) | 637 | 3:30:47 | 2025/10/19 |
| ((Glaucoma[MeSH Terms]) OR (Glaucomas)) AND ((((((((((((Cataract[MeSH Terms]) OR (Cataracts)) OR (Lens Opacities)) OR (Lens Opacity)) OR (Opacities, Lens)) OR (Opacity, Lens)) OR (Cataract, Membranous)) OR (Cataracts, Membranous)) OR (Membranous Cataract)) OR (Membranous Cataracts)) OR (Pseudoaphakia)) OR (Pseudoaphakias)) | | | ("glaucoma"[MeSH Terms] OR ("glaucoma"[MeSH Terms] OR "glaucoma"[All Fields] OR "glaucomas"[All Fields] OR "glaucoma s"[All Fields])) AND ("cataract"[MeSH Terms] OR ("cataract"[MeSH Terms] OR "cataract"[All Fields] OR "cataracts"[All Fields] OR "cataractic"[All Fields] OR "cataractous"[All Fields]) OR ("cataract"[MeSH Terms] OR "cataract"[All Fields] OR ("lens"[All Fields] AND "opacities"[All Fields]) OR "lens opacities"[All Fields]) OR ("cataract"[MeSH Terms] OR "cataract"[All Fields] OR ("lens"[All Fields] AND "opacity"[All Fields]) OR "lens opacity"[All Fields]) OR ("cataract"[MeSH Terms] OR "cataract"[All Fields] OR ("opacities"[All Fields] AND "lens"[All Fields]) OR "opacities lens"[All Fields]) OR ("cataract"[MeSH Terms] OR "cataract"[All Fields] OR ("opacity"[All Fields] AND "lens"[All Fields]) OR "opacity lens"[All Fields]) OR ("cataract"[MeSH Terms] OR "cataract"[All Fields] OR ("cataract"[All Fields] AND "membranous"[All Fields]) OR "cataract membranous"[All Fields]) OR ("cataract"[MeSH Terms] OR "cataract"[All Fields] OR ("cataracts"[All Fields] AND "membranous"[All Fields])) OR ("cataract"[MeSH Terms] OR "cataract"[All Fields] OR ("membranous"[All Fields] AND "cataract"[All Fields]) OR "membranous cataract"[All Fields]) OR ("cataract"[MeSH Terms] OR "cataract"[All Fields] OR ("membranous"[All Fields] AND "cataracts"[All Fields]) OR "membranous cataracts"[All Fields]) OR ("cataract"[MeSH Terms] OR "cataract"[All Fields] OR "pseudoaphakia"[All Fields]) OR ("cataract"[MeSH Terms] OR "cataract"[All Fields])) | 14,146 | 3:29:01 | 2025/10/19 |
| ((((Trabectome) OR (ab interno)) OR (ab externo)) OR (triple procedure)) OR (Phacotrabeculectomy) | | | "Trabectome"[All Fields] OR (("abnormalities"[MeSH Subheading] OR "abnormalities"[All Fields] OR "ab"[All Fields]) AND "interno"[All Fields]) OR (("abnormalities"[MeSH Subheading] OR "abnormalities"[All Fields] OR "ab"[All Fields]) AND "externo"[All Fields]) OR (("triple"[All Fields] OR "triples"[All Fields]) AND ("methods"[MeSH Terms] OR "methods"[All Fields] OR "procedure"[All Fields] OR "methods"[MeSH Subheading] OR "procedures"[All Fields] OR "procedural"[All Fields] OR "procedurally"[All Fields] OR "procedure s"[All Fields])) OR ("phacotrabeculectomies"[All Fields] OR "phacotrabeculectomy"[All Fields]) | 55,505 | 3:28:31 | 2025/10/19 |
| Phacoemulsification | |  | "phacoemulsification"[MeSH Terms] OR "phacoemulsification"[All Fields] OR "phacoemulsifications"[All Fields] OR "phakoemulsification"[All Fields] | 17,671 | 3:27:06 | 2025/10/19 |
| (((((((((((Cataract[MeSH Terms]) OR (Cataracts)) OR (Lens Opacities)) OR (Lens Opacity)) OR (Opacities, Lens)) OR (Opacity, Lens)) OR (Cataract, Membranous)) OR (Cataracts, Membranous)) OR (Membranous Cataract)) OR (Membranous Cataracts)) OR (Pseudoaphakia)) OR (Pseudoaphakias) | | | "cataract"[MeSH Terms] OR ("cataract"[MeSH Terms] OR "cataract"[All Fields] OR "cataracts"[All Fields] OR "cataractic"[All Fields] OR "cataractous"[All Fields]) OR ("cataract"[MeSH Terms] OR "cataract"[All Fields] OR ("lens"[All Fields] AND "opacities"[All Fields]) OR "lens opacities"[All Fields]) OR ("cataract"[MeSH Terms] OR "cataract"[All Fields] OR ("lens"[All Fields] AND "opacity"[All Fields]) OR "lens opacity"[All Fields]) OR ("cataract"[MeSH Terms] OR "cataract"[All Fields] OR ("opacities"[All Fields] AND "lens"[All Fields]) OR "opacities lens"[All Fields]) OR ("cataract"[MeSH Terms] OR "cataract"[All Fields] OR ("opacity"[All Fields] AND "lens"[All Fields]) OR "opacity lens"[All Fields]) OR ("cataract"[MeSH Terms] OR "cataract"[All Fields] OR ("cataract"[All Fields] AND "membranous"[All Fields]) OR "cataract membranous"[All Fields]) OR ("cataract"[MeSH Terms] OR "cataract"[All Fields] OR ("cataracts"[All Fields] AND "membranous"[All Fields])) OR ("cataract"[MeSH Terms] OR "cataract"[All Fields] OR ("membranous"[All Fields] AND "cataract"[All Fields]) OR "membranous cataract"[All Fields]) OR ("cataract"[MeSH Terms] OR "cataract"[All Fields] OR ("membranous"[All Fields] AND "cataracts"[All Fields]) OR "membranous cataracts"[All Fields]) OR ("cataract"[MeSH Terms] OR "cataract"[All Fields] OR "pseudoaphakia"[All Fields]) OR ("cataract"[MeSH Terms] OR "cataract"[All Fields]) | 94,551 | 3:26:28 | 2025/10/19 |
| (Glaucoma[MeSH Terms]) OR (Glaucomas) | | | "glaucoma"[MeSH Terms] OR "glaucoma"[MeSH Terms] OR "glaucoma"[All Fields] OR "glaucomas"[All Fields] OR "glaucoma s"[All Fields] | 92,479 | 3:23:58 | 2025/10/19 |
