# Supplementary figures and images for: Combined phacoemulsification and angle filtering procedures versus phacoemulsification with clinical outcomes in primary glaucoma coexisting with cataracts: a meta-analysis of randomized controlled trials
Source: Front Ophthalmol (Lausanne). 2026 Jun 8;6:1787044. doi: 10.3389/fopht.2026.1787044 (PMC13283822; doi:10.3389/fopht.2026.1787044)

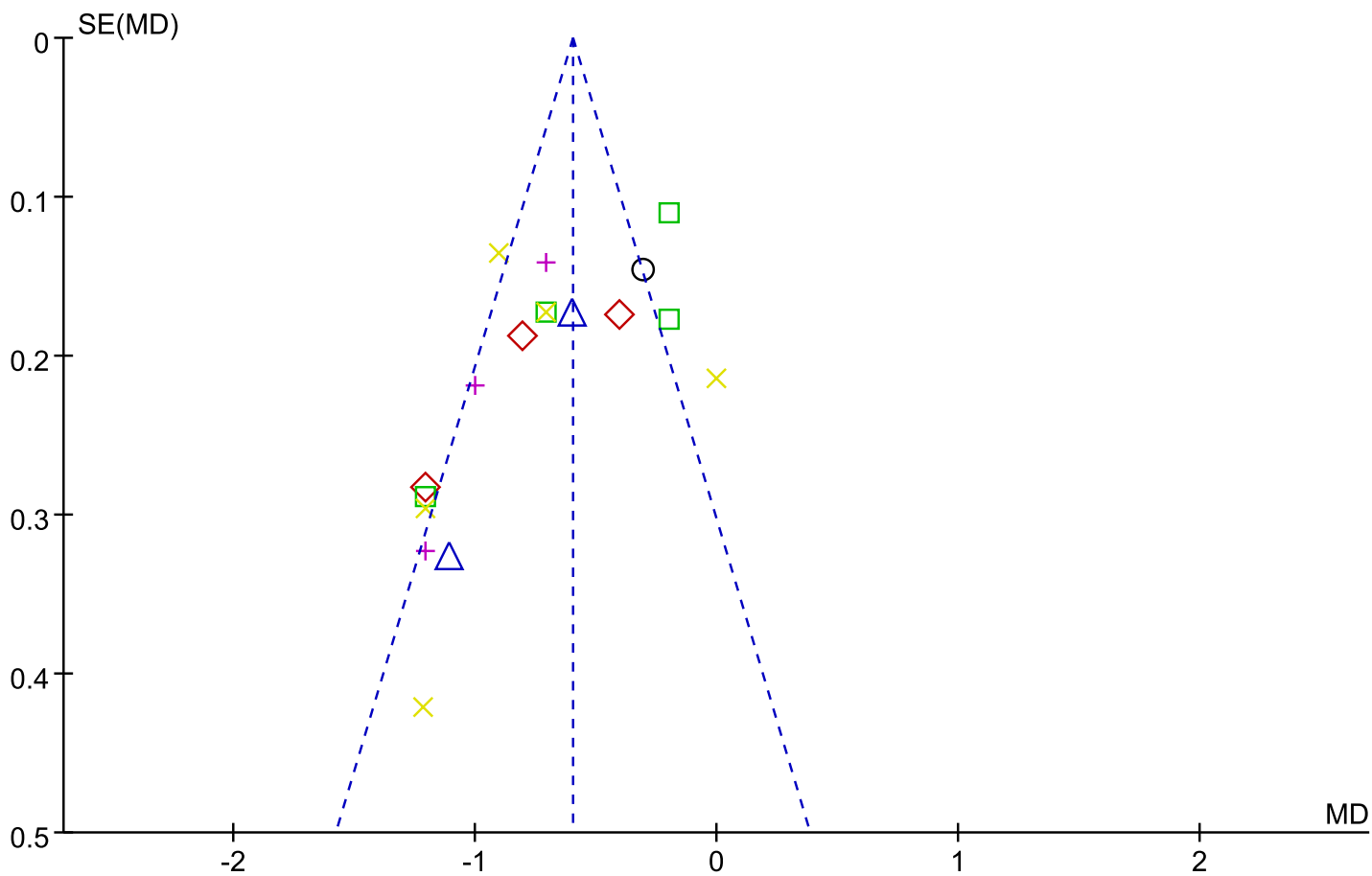

**Subgroups**

|             |             |             |             |              |              |
|-------------|-------------|-------------|-------------|--------------|--------------|
| ○ 1st month | ◇ 3rd month | □ 6th month | △ 9th month | × 12th month | + 24th month |
|-------------|-------------|-------------|-------------|--------------|--------------|

Supplement: Supplementary file 2 [file SupplementaryFile2.pdf]

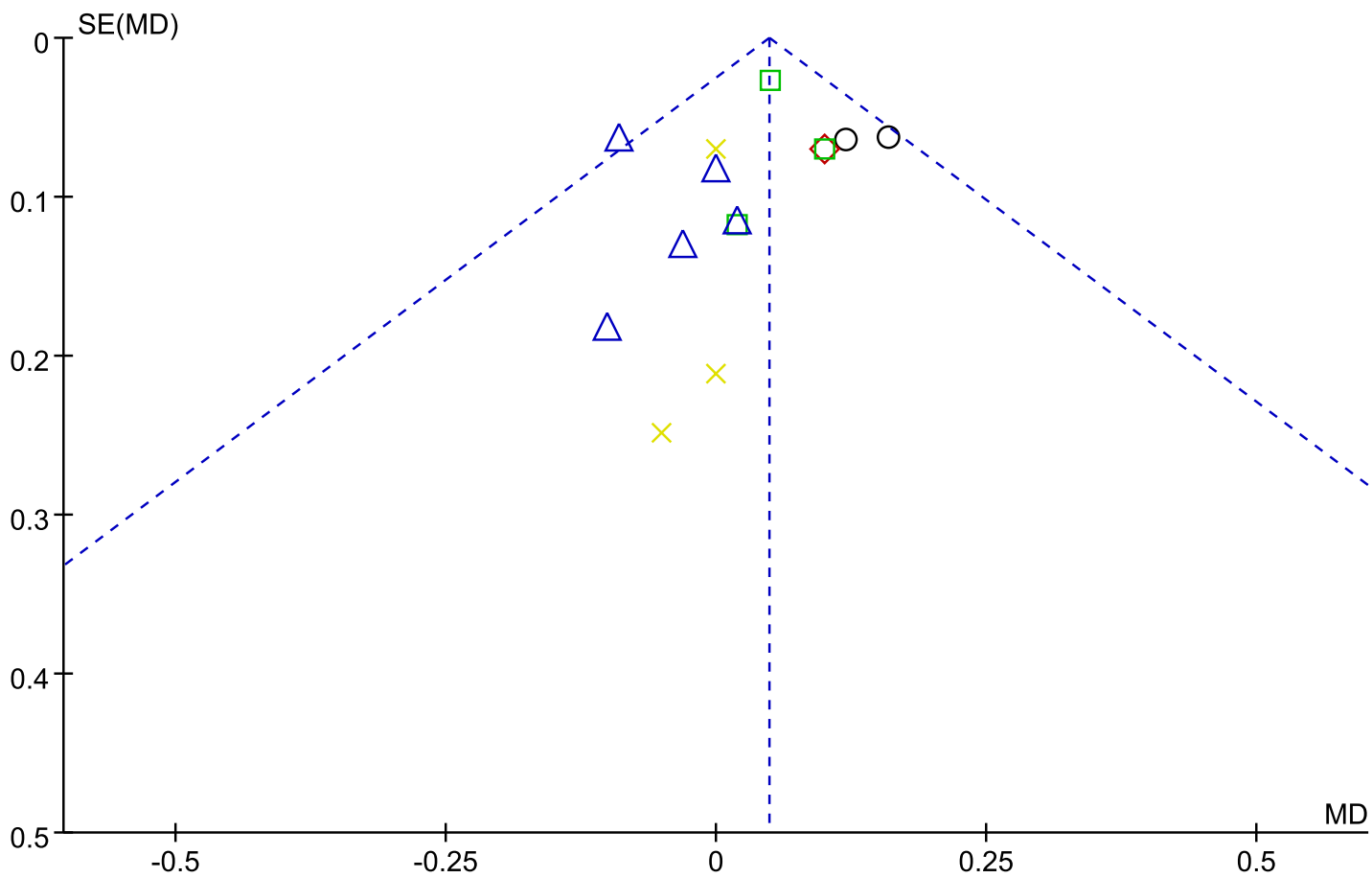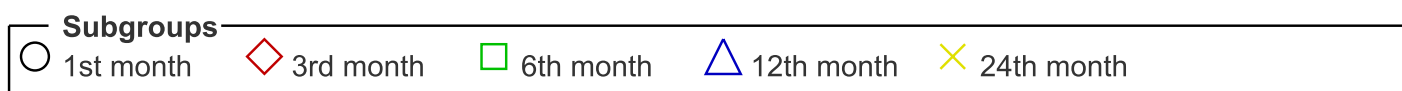

Supplement: Supplementary file 3 [file SupplementaryFile3.pdf]

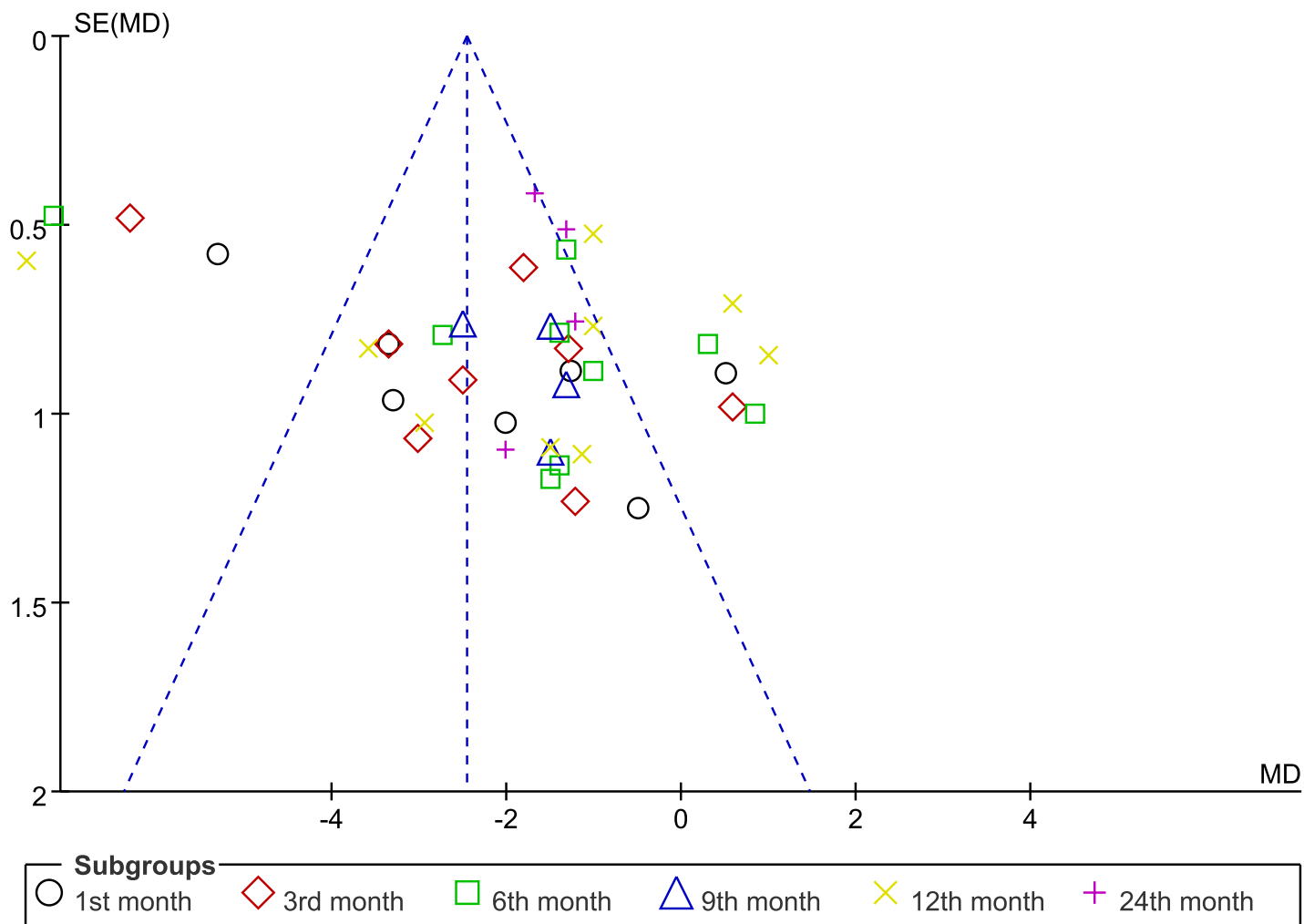

Supplement: Supplementary file 4 [file SupplementaryFile4.pdf]
