# Supplementary material for: Combined phacoemulsification and angle filtering procedures versus phacoemulsification with clinical outcomes in primary glaucoma coexisting with cataracts: a meta-analysis of randomized controlled trials
Source: Front Ophthalmol (Lausanne). 2026 Jun 8;6:1787044. doi: 10.3389/fopht.2026.1787044 (PMC13283822; doi:10.3389/fopht.2026.1787044)
Supplement: Supplementary file 5 [file SupplementaryFile5.pdf]

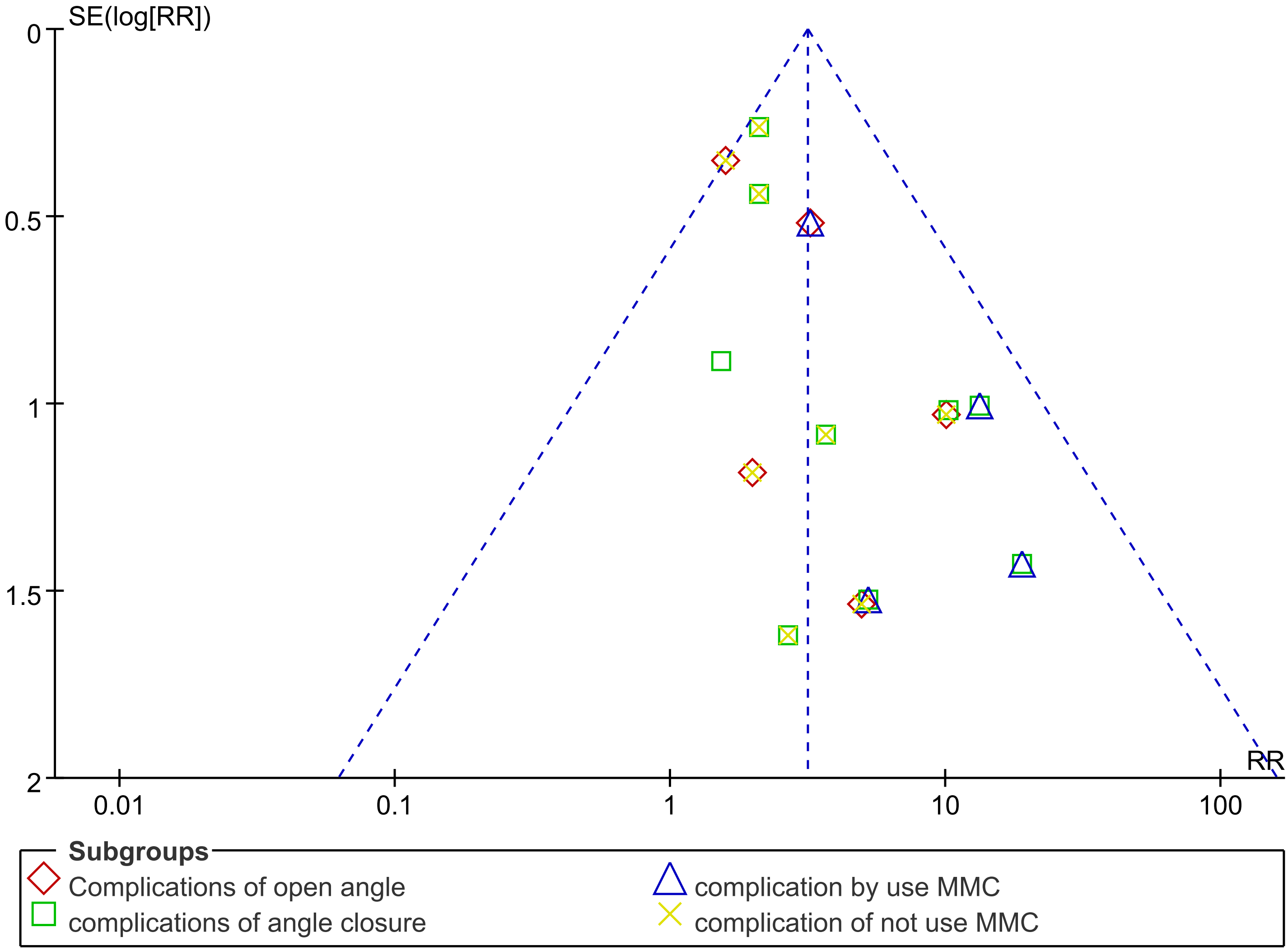
SE(log[RR])
0
0.5
1
1.5
2
0.01
0.1
1
10
100
RR
Subgroups
Complications of open angle
complications of angle closure
complication by use MMC
complication of not use MMC
